# Heterochirality results from reduction of maternal *diaph* expression in a terrestrial pulmonate snail

**DOI:** 10.1186/s40851-018-0120-0

**Published:** 2019-01-10

**Authors:** Takeshi Noda, Noriyuki Satoh, Takahiro Asami

**Affiliations:** 1 Department of Biology, Faculty of Sciences, Shinshu University, Matsumoto, 390-8621 Japan; 2 Marine Genomics Unit, Okinawa Institute of Science and Technology Graduate University, Onna, Okinawa, 904-0495 Japan

**Keywords:** Spiral cleavage, Left-right asymmetry, Gene duplication, *Bradybaena*, Pulmonate, Gastropod

## Abstract

**Background:**

Spiral cleavage is a feature of non-ecdysozoan protostomes, in which left-right reversal frequently evolved in gastropod molluscs. In pulmonate gastropods, maternal molecules are responsible for chirality patterning, on which the polarities of visceral and coiling asymmetries depend. In the pond snail, *Lymnaea stagnalis* (the clade Hygrophila), a frame-shift mutation of one of tandem-duplicated, diaphanous-related formin genes (*diaph*) resulted in incomplete reversal from dextral to sinistral cleavage. Is this mechanism of chirality regulation common to, or shared with other pulmonates? To answer this question, we examined genes involved in chirality patterning in the land snail, *Bradybaena similaris* which belongs to the clade Stylommatophora.

**Results:**

Both dextral and sinistral siblings develop from progeny of a racemic mutant of *B. similaris*. Differences in maternal mRNAs between the two strains were searched by transcriptome analyses. We found fifty maternal transcripts that exhibited less expression in early embryos of the mutant strain. The most conspicuous was a homolog of *diaph.* The *diaph* gene was duplicated in the stylommatophoran ancestor (*diaph-a* and *diaph-b*), as in the case of the ancestor of Lymnaea (*Lsdiaph1* and *Lsdiaph2*). The quantity of maternal *diaph-b* mRNA was drastically reduced in early embryos of the racemic mutant compared to wild-type, while *diaph-a* expression was at nearly the same level in both strains. Unlike the case of *Lsdiaph2*, which is frame-shifted to produce truncated peptides in the mutant of *L. stagnalis*, however, *Bsdiaph-b* mRNA in the mutant strain is not frame-shifted and most probably produces normal Diaph-b protein. These results suggest the presence of regulatory mechanisms of gene expression for chirality patterning in pulmonate gastropods, although genomic analyses are required for confirmation.

**Conclusions:**

Heterochirality resulting from the loss of polarity control in spiral cleavage does not require mutation of the *diaph* gene in *B. similaris*. The determination of left-right polarity instead depends on the expression of this *diaph* gene, which is duplicated in stylommatophoran *Bradybaena*, as well as in hygrophilan *Lymnaea*. Our results provide an avenue to identifying a regulatory mechanism that controls the direction of spiral cleavage in gastropods.

**Electronic supplementary material:**

The online version of this article (10.1186/s40851-018-0120-0) contains supplementary material, which is available to authorized users.

## Background

Metazoans exhibit different modes of cleavage, including radial (starfish), spiral (snails), and bilateral (tunicates) cleavage (e.g., [1] Gilbert and Raunio, 1997; [2] Nielsen, 2012). Spiral cleavage is a characteristic for several taxa of non-ecdysozoan protostomes, represented by molluscs and annelids, and is recognized as an embryological feature of these animal groups (lophotrochozoans or spiralians), although the “Spiralia” vs “Lophotrochozoa” nomenclature has been debated (e.g., [3] Dunn et al., 2008 [4] Henry, 2014; [5] Laumer et al., 2015). In gastropod molluscs, the left-right polarity of spiral cleavage makes the blastomere asymmetric. The left-right geometry of blastomeres by itself regulates the handedness of subsequent zygotic gene expression, so as to develop into a clockwise-coiled (dextral) or counterclockwise-coiled (sinistral) snail ([6] Kuroda et al., 2009).

Maternal inheritance ([7] Toyama, 1913) of left-right reversal by nuclear gene mutation in pulmonates, which are hermaphrodites, was discovered through a breeding experiment with the dextral pond snail *Lymnaea peregra* ([8] Boycott and Driver, 1923, [9] Sturtevant, 1923). Mutant hatchlings exhibited reversal in bilateral visceral asymmetry as well as coiling direction. Transplantation experiments of egg cytoplasm between the wild-type dextral and mutant sinistral strains of *L. peregra* demonstrated the presence of maternally supplied information that is involved in patterning of the spiral cleavage ([10] Freeman and Lundelius, 1982). In *L. stagnalis*, progeny of a wild-type mother (*DD* or *Ds*) exhibit dextral cleavage while those of a mutant (*ss*) display incomplete sinistral cleavage ([11] Asami et al., 2008, [12] Utsuno et al. 2011). In efforts to discover the maternally expressed genes responsible for patterning the chirality ([13] Hierck et al., 2005, [14] Shibazaki et al., 2004, [15] Harada et al., 2004), Davison et al. [16] and Kuroda et al. [17] independently succeeded in identifying diaphanous-related formin (the homolog of human *diaph1, diaph2*, and *diaph3*) as a candidate gene associated with the direction of left-right asymmetry in *L. stagnalis.* In this species, *diaph* is duplicated into two copies, one of which became frame-shifted in mutants. Viable progeny of the mutant homozygote (*ss*) develop into the sinistral form, while most siblings fail to develop ([12] Utsuno et al. 2011). Thus, these maternally expressed genes may be responsible for symmetry breaking in the Hygrophila, which is one of the two clades dividing the Pulmonata.

Left-right reversed groups/species have frequently evolved in gastropods, especially in pulmonates, among the Lophotrochozoa/Spiralia ([18] Okumura et al., 2008, [19] Gittenberger et al., 2012). In each phylogenetically independent event, a breeding population must have been fixed for a normally viable mutant allele for reversal. For example, in the Stylommatophora, the sister clade to the Hygrophila, populations repeatedly fixed for reversal in *Euhadra* ([20] Uesima and Asami, 2003) and *Satsuma* ([21] Hoso et al. 2010) of the mainly dextral Camaenidae as well as in *Albinaria* of the mainly sinistral Clausiliidae ([22] Kornilios et al., 2015). Within the Hygrophila, *diaph* is duplicated in the dextral *Lymnaea*, but not in the sinistral sister groups, *Physella acuta* or *Indoplanorbis exustus*e ([16] Davison et al., 2016, Noda et al., unpublished data). The commonality and diversity of genic mechanisms responsible for these recurrent evolutions of reversal remain unclear.

The racemic mutant of the dextral land snail *Bradybaena similaris* produces both dextral and sinistral progeny. This racemism provides a unique opportunity to examine the genic mechanism for the regulation of left-right polarity of spiral cleavage in the Camaenidae. A reverse-coiled mutant can rarely reproduce because of physical difficulty in mating with the wild type even if it was found in the wild, especially in case of simultaneously reciprocally copulating stylommatophorans such as camaenids ([20] Ueshima and Asami, 2003; [23] Asami et al. 1998). We however discovered a wild individual that was phenotypically dextral, but homozygous for the racemic allele, which made it possible to establish a racemic strain of *B. similaris* ([24] Utsuno and Asami, 2010). The mutant strains show maternal inheritance for chirality with genetically recessive alleles, which relies on maternal storage of a gene product that promotes dextral cleavage in the wild type. The racemism suggests that this phenotype may results from a loss-of-function mutation for determination of left-right polarity in spiral cleavage. By taking advantage of this chirality-mutant strain, only available in the Stylommatophora, we carried out RNA-seq analyses to test whether the racemic mutant exhibits a transcriptional defect.

Here we show that the maternal expression of one of the *diaph*-homologs is conspicuously reduced in the progeny of the racemic mutant of *B. similaris* with no mutation in the gene itself. Our results open a new ground to explore a regulatory mechanism that controls the left-right polarity of development in gastropods.

## Materials and methods

### Biological materials

**Fig. 1:**
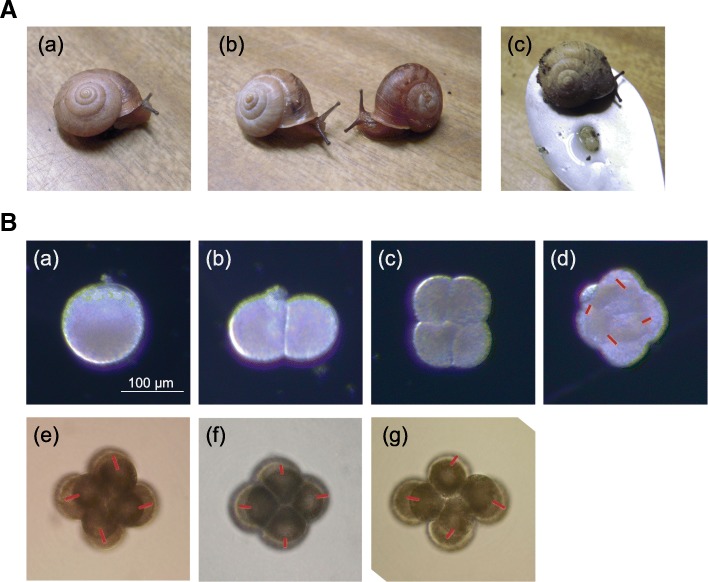
(**a**) Adults of *Bradybaena similaris.* (a) Wild *B. similaris* (dextral). (b) Racemic mutants of *B. similaris*. Dextral (left) and sinistral (right) ones. Wild-type strain that produces clockwise-coiled (dextral) snails and a mutant strain that produces either clockwise or counterclockwise-coiled (sinistral) snails (right). (c) *B. similaris* lays several tens eggs during a period of several hours. (**b**) Embryos of *B. similaris*. (a)-(d) Embryos from a wild individual. (e)-(g) Embryos from a racemic mutant individual. (a) egg, (b) 2-cell, (c) 4-cell, (d)-(g) 8-cell. Cleavage directions are shown by red lines. Cells divide clockwise direction (d) in wild 4-cell embryos from animal pole view, while clockwise to counterclockwise direction (e)-(g) in racemic mutant ones

The land snail *Bradybaena similaris*, was used in this study. Snails of the dextral wild type and racemic mutant were collected from Kashiwa, Chiba, Japan and have been maintained in our laboratory ([24] Utsuno and Asami, 2010). Wild-type snails exhibit the clockwise-coiled (dextral) phenotype (Fig. 1A-a) whereas the progeny of the racemic mutant includes both clockwise- and counterclockwise-coiled (sinistral) siblings (Fig. 1A-b). Snails of the Pulmonata, including *B. similaris*, are hermaphroditic. Their fertilization occurs internally through simultaneous reciprocal copulation with the other individual with the same chiral phenotype.

The direction of spiral cleavage is determined maternally. Genetic studies showed that offspring of a *++* or *+ r* mother all become dextral individuals. On the other hand, *rr* homozygosity has variable effects on embryogenesis, and usually result in 70% dextral and 30% sinistral offspring ([24] Utsuno and Asami, 2010). Females usually lay several tens of eggs over a period of several hours (Fig. 1A-c). This egg laying behavior makes difficult to collect enough eggs before the initiation of cleavage. We sampled early-embryos from the 1-cell to 16-cell stages for analysis of maternal genes. Although it is not known when maternal to zygotic transition initiates in *B. similaris*, 16-cell stage is the first stage for transcription in another gastropod species, *Lymnaea*([25] Biggelaar, 1971). Two sets of RNA-seq analyses were carried out using approximately 100 early embryos each from six to eight clutches. In addition, juveniles and digestive glands of adults were collected to examine their gene expression profiles.

### RNA-seq, analyses of differential gene expression, mRNA quantification and molecular phylogeny

Total RNA was extracted using a standard TRIzol protocol procedure (Thermo Fisher Scientific), and cDNA libraries were prepared using a TruSeq RNA Library Prep Kit v2 (Illumina). RNA quality was checked with an Agilent Technologies 2100 Bioanalyzer using an Agilent RNA 6000 Nano Kit. Sequencing was performed using an Illumina Hiseq4000 and MiSeq. De novo assembly of whole RNA sequence reads was performed using a de Bruijn graph-based program, Trinity-v2.3.2 ([26] Grabherr et al., 2011; [27] Haas et al., 2013). All Illumina reads are available from NCBI database under accession nos. SRR804510–SRR804517.

Quantification of transcripts was carried out by software tools for expression analysis, Tophat 2.1.1 and Cufflinks 2.2.1([28] Trapnell et al., 2012) using RNA-seq results and the gene models constructed by Trinity. Differentially expressed gene analysis employed EdgeR 3.22.5 ([29] Robinson et al., 2010) with the mapping results from Tophat. Differentially expressed genes were annotated using Blastx against reference protein sequences of *Homo sapiens* and genome sequences decoded in the mollusc, *Biomphalaria glabrata,* in the NCBI database*.* Quantification by qRT-PCR was also carried out as previously described with slight modifications ([30] Noda, 2011). Total RNA from batches of early embryos (egg to 16-cell stage) was extracted by standard TRIzol protocol. Three each of samples were prepared from siblings of wild, racemic and F1 strains. cDNA reverse transcribed from 10 ng total RNA was used for each qRT-PCR reaction with step one plus real time PCR system (step one software version 2.3) by the standard curve methods. Sequences of primers are as follows; *Bsidiaph-b* forword; TCAAAGACTGTGATTGGCTGA, *Bsidiaph-b* reverse; GCTCAGAGAATTCATGAGTACCG, *Bsidiaph-a* forword; CCATGAAGCTTCCGTTTGAT, *Bsidiaph-a* reverse; TTCATGTCATCTGGCTCTGG. We quantified all splicing variants of *Bsdiaph-a* together because of technical difficulty to distinguish *Bsdiaph-a-× 1* and *Bsdiaph-a-× 2* for quantification, including the construction of specific primers to each variant for qRT-PCR.

Molecular phylogenetic analysis was carried out with MrBayes version 3.2.6, as previously described with slight modifications ([30] Noda, 2011). Amino acid sequences of conserved FH2 domains were aligned by ClustalW version 2.1.0 for constructing the molecular phylogenetic tree. LG model was adopted as an evolutionary model. List of sequence IDs are available in Additional file 1: Table S2.

All informatic analyses were carried out using default parameters.

## Results

### Identification of differentially-expressed maternal genes in wild and mutant strains

Total RNA-seq reads obtained for wild-1embryos, wild-2 embryos, mutant-1 embryos, mutant-2 embryos, wild juveniles, and mutant juveniles, wild digestive gland, and mutant digestive gland were 24,192,382, 27,239,716, 30,957,316, 39,046,157, 29,971,051, 36,976,111, 30,075,674, and 31,158,448, respectively (Additional file 2). After Trinity treatment, reads were assembled to 590,901 contigs (Additional file 2). The N50 of the assembled transcriptomes was 829 nucleotides (nts) (the longest being 31,291 nts). RNA-seq reads were mapped to these contigs using Tophat for the differentially expressed gene analysis and mRNA quantification.

Comparisons using the EdgeR method ([29] Robinson et al., 2010; [31] McCarthy et al., 2012) of the maternal transcriptomes between wild-type and mutant strains (*p*-value <1e-30) demonstrated 50 genes that were significantly higher in the former than in the latter (Table 1). BlastX search against the *Homo sapiens* genome and that of a gastropod, *Biomphalaria glabrata,* was carried out to determine the similarity of transcripts to known proteins listed in the NCBI database. Of those, 34 transcripts showed similarity to proteins within the database, while another 16 showed no sequence similarity (Table 1). The transcript that was most reduced in mutant strains encodes a homolog of human *DIAPH2* (Table 1, TRINITY_DN171781_c1_g2, *p*-value from EdgeR; 7.34E-158, LogFc; 6.26). So, we named the gene as *Bsdiaph* (*B. similaris* gene for Diaph protein). The reads of *Bsdiaph* transcript were abundant in early embryos of the wild-type (3933 reads in Exp-1 and 4207 in Exp-2) and very few in early embryos of mutants (49 reads in Exp-1 and 57 in Exp-2). This result suggests that maternal expression of *diaph* is greatly suppressed in the *B. similaris* mutant strain, in which embryogenesis produced racemic (dextral and sinistral) progeny.

**Table 1:** Top 50 transcripts differentially expressed in wild-type and mutant-type strains

ID	Wild1	Wild2	Mut1	Mut2	LogFC	p.value	q.value	H.sapBlastx	E.value	H.sapGene	B.glaBlastx	E.value
TRINITY_DN171781_c1_g2	3933	4207	49	57	6.26	7.34E-158	3.36E-152	NP_006720.1	1.00E-118	DIAPH2	XP_013077820.1	0
TRINITY_DN144442_c0_g1	977	1005	2	1	9.22	8.36E-116	4.25E-111	no hit			XP_013090645.1	9.00E-14
TRINITY_DN179541_c2_g2	522	607	19	21	4.81	4.21E-79	8.37E-75	XP_005251163.1	2.00E-11	RIMS2	XP_013060759.1	1.00E-83
TRINITY_DN158236_c0_g1	481	550	16	14	5.1	7.88E-77	1.39E-72	NP_009126.2	2.00E-118	POLI	XP_013083393.1	9.00E-101
TRINITY_DN162618_c0_g1	884	824	13	20	5.69	1.58E-74	2.59E-70	no hit			no hit	
TRINITY_DN178056_c0_g1	640	601	4	9	6.58	4.32E-74	6.81E-70	no hit			XP_013087004.1	4.00E-22
TRINITY_DN174403_c0_g1	277	327	5	4	6.07	3.17E-68	3.37E-64	XP_011531514.1	9.00E-09	TDRD15	XP_013081149.1	1.00E-32
TRINITY_DN169314_c0_g5	436	513	21	18	4.6	8.03E-67	7.35E-63	NP_000093.1	2.00E-44	CYP17A1	XP_013081916.1	5.00E-174
TRINITY_DN184104_c10_g9	750	745	47	63	3.76	5.35E-57	2.66E-53	no hit			no hit	
TRINITY_DN184104_c11_g1	5022	4966	287	436	3.79	6.70E-56	3.23E-52	no hit			no hit	
TRINITY_DN159907_c0_g1	714	1107	30	37	4.76	1.73E-55	7.91E-52	no hit			XP_013087987.1	0
TRINITY_DN183369_c0_g1	222	250	6	9	4,98	9.51E-55	4.26E-51	XP_016864717.1	2.00E-20	FBN2	XP_013063637.1	0
TRINITY_DN158557_c0_g7	169	210	2	2	6.43	4.61E-54	1.93E-50	no hit			no hit	
TRINITY_DN179544_c0_g9	534	556	48	57	3.37	2.28E-52	8.42E-49	no hit			no hit	
TRINITY_DN167348_c0_g1	166	193	1	5	5.9	6.08E-51	2.08E-47	no hit			XP_013079795.1	1.00E-05
TRINITY_DN174045_c0_g5	398	434	11	26	4.49	8.54E-51	2.89E-47	NP_036566.1	3.00E-79	SLC17A5	XP_013064858.1	5.00E-128
TRINITY_DN183989_c2_g7	140	165	2	0	7.31	7.08E-50	2.25E-46	NP_001309433.1	1.00E-30	ADRA1A	XP_013094752.1	0
TRINITY_DN170834_c5_g1	249	220	0	3	7.29	8.08E-50	2.55E-46	NP_001792.2	4.00E-71	CDO1	XP_013094819.1	5.00E-48
TRINITY_DN177866_c2_g1	491	522	50	62	3.17	1.56E-49	4.80E-46	XP_016881871.1	1.00E-109	ZNF569	XP_013062762.1	2.00E-103
TRINITY_DN184025_c3_g1	689	619	31	24	4.57	2.31E-48	6.69E-45	no hit			no hit	
TRINITY_DN159941_c9_g1	138	173	3	0	6.7	1.62E-47	4.42E-44	no hit			XP_013070287.1	5.00E-07
TRINITY_DN183351_c2_g3	178	230	0	7	5.87	2.85E-47	7.63E-44	NP_004470.1	6.00E-33	FUT7	XP_013095063.1	9.00E-91
TRINITY_DN181463_c0_g5	510	507	23	43	3.95	2.93E-44	6.47E-41	XP_016872937.1	5.00E-37	FOLH1	XP_013096505.1	6.00E-72
TRINITY_DN181101_c5_g2	108	136	0	0	–	6.44E-44	1.40E-40	no hit			no hit	
TRINITY_DN172756_c1_g2	300	340	33	36	3.21	1.55E-43	3.31E-40	no hit			XP_013061254.1	4.00E-04
TRINITY_DN168064_c0_g2	376	401	30	21	3.93	1.79E-43	3.80E-40	no hit			XP_013080707.1	6.00E-05
TRINITY_DN161165_c0_g1	193	231	13	16	3.87	5.92E-43	1.21E-39	NP_942592.1	3.00E-06	ANKRD17	XP_013077829.1	3.00E-08
TRINITY_DN173891_c0_g1	105	149	0	1	7.99	2.61E-41	4.67E-38	NP_003050.2	1.00E-52	SLC22A4	XP_013084471.1	7.00E-180
TRINITY_DN174950_c1_g3	101	107	0	1	7.7	5.18E-40	8.58E-37	NP_001010908.1	2.00E-03	C1QL3	no hit	
TRINITY_DN165372_c0_g2	141	190	0	7	5.56	7.50E-40	1.23E-36	no hit			no hit	
TRINITY_DN167428_c4_g1	210	265	20	22	3.5	2.87E-39	4.59E-36	XP_016884664.1	0.01	LOC105373057	no hit	
TRINITY_DN165835_c0_g4	96	103	0	0	–	9.32E-39	1.44E-35	no hit			no hit	
TRINITY_DN175874_c1_g2	88	116	0	0	–	4.01E-38	6.01E-35	no hit			no hit	
TRINITY_DN173785_c0_g1	90	97	0	0	–	3.08E-37	4.28E-34	NP_000021.1	2.00E-118	AGXT	XP_013088624.1	1.00E-177
TRINITY_DN175279_c0_g4	169	214	16	16	3.58	1.22E-35	1.53E-32	no hit			no hit	
TRINITY_DN173815_c2_g1	236	309	30	35	3.07	4.85E-35	5.86E-32	no hit			XP_013086107.1	7.00E-09
TRINITY_DN181712_c0_g1	315	330	43	40	2.96	6.01E-35	7.19E-32	no hit			no hit	
TRINITY_DN153826_c0_g2	171	216	6	17	4.07	7.05E-35	8.40E-32	no hit			no hit	
TRINITY_DN167777_c0_g1	299	316	36	51	2.82	4.08E-34	4.64E-31	NP_056066.2	1.00E-12	ATMIN	XP_013087469.1	2.00E-19
TRINITY_DN171245_c0_g1	862	933	196	242	2.03	6.88E-34	7.58E-31	XP_011530689.1	2.00E-11	RRSS12	XP_013062786.1	0
TRINITY_DN174413_c0_g1	620	681	104	147	2.37	1.31E-33	1.38E-30	no iht			no hit	
TRINITY_DN157698_c0_g2	316	309	23	40	3.31	1.53E-33	1.60E-30	no hit			no hit	
TRINITY_DN183119_c0_g1	201	206	21	23	3.21	4.93E-33	4.99E-30	no hit			XP_013085093.1	1.00E-167
TRINITY_DN178439_c0_g1	1438	1471	172	271	2.72	3.54E-32	3.39E-29	XP_011532070.1	4.00E-121	CNTN3	XP_013078877.1	0
TRINITY_DN182175_c0_g2	246	304	31	47	2.82	6.26E-32	5.86E-29	NP_001307550.1	2.00E-39	GRIK1	XP_013084907.1	3.00E-138
TRINITY_DN175846_c1_g1	138	197	11	13	3.83	2.54E-31	2.27E-28	NP_061951.1	5.00E-44	UGT1A5	XP_013061108.1	1.00E-92
TRINITY_DN164333_c2_g2	312	360	39	26	3.7	2.73E-31	2.43E-28	no hit			no hit	
TRINITY_DN169755_c2_g1	29	30	2	6	2.9	2.99E-31	2.64E-28	no hit			XP_013084581.1	3.00E-109
TRINITY_DN182882_c0_g2	111	129	6	10	3.91	4.56E-31	3.99E-28	XP_011507944.1	8.00E-84	PLA2G4A	XP_013068547.1	6.00E-140
TRINITY_DN183681_c4_g1	117	174	4	9	4.48	5.29E-31	4.59E-28	NP_001128360.1	5.00E-21	LOC106073088	XP_013089011.1	4.00E-50

The next four transcripts with sequence similarity to human proteins corresponded to RIMS2 (TRINITY_DN179541_c2_g2), POL1 (TRINITY_DN158236_c0_g1), TDRD15 (TRINITY_DN174403_c0_g1) and CYP17A1 (TRINITY_DN169314_c0_g5_i2) (Table 1).

TRINITY_DN158236_c0_g1 is an orthologue of human POLI, which is an error-prone DNA polymerase involved in DNA repair. Genomes of *Homo sapiens*, *Biomophalalia glablata* and *Aplysia californica* and our transcriptome data contain each one POLI orthologue. TRINITY_DN184104_c10_g9 is a homologue of human CYP17A1, which is a member of the cytochrome P450 superfamily of enzymes for metabolic reactions, however, orthologous relationship with human genes was not clear because of highly diversification of these genes. Genomes of *Biomophalalia glablata* and *Aplysia californica* contain each one orthologue of TRINITY_DN184104_c10_g9. The homology between human RIMS2 and TDRD15 and *Bradybaena* transcripts (TRINITY_DN179541_c2_g2 and TRINITY_DN174403_c0_g1) was not clear, because their similarity was limited to small protein motifs or domains. These genes were highly diversified or independent in the Pulmonata lineage. Further characterization of genes with weak expression in racemic mutant embryos is the focus of our next investigation.

### Identification and characterization of *diaph-a* and *diaph-b* transcripts in *Bradybaena similaris*

In *L. stagnalis, diaph* duplicated into *Lsdiaph1* and *Lsdiaph2,* which are tandemly aligned in the genome. *Lsdiaph1* and *Lsdiaph2* encode diaphanous-related formin proteins composed of approximately 1100 amino acids. Proteins LsDiaph1 and LsDiaph2 have 89.4% identity at the amino acid sequence level ([16] Kuroda et al. 2016). Davidson et al. (2016) described the mutated gene as *ldia2* (*Lsdia2),* while Kuroda et al. (2016) called it *Lsdia1*. Based on an assumption that the original (ancestral) one is molecularly more conserved than the duplicated counterpart, we here followed the naming of the gene by Davidson et al. (2016).

**Fig. 2:**
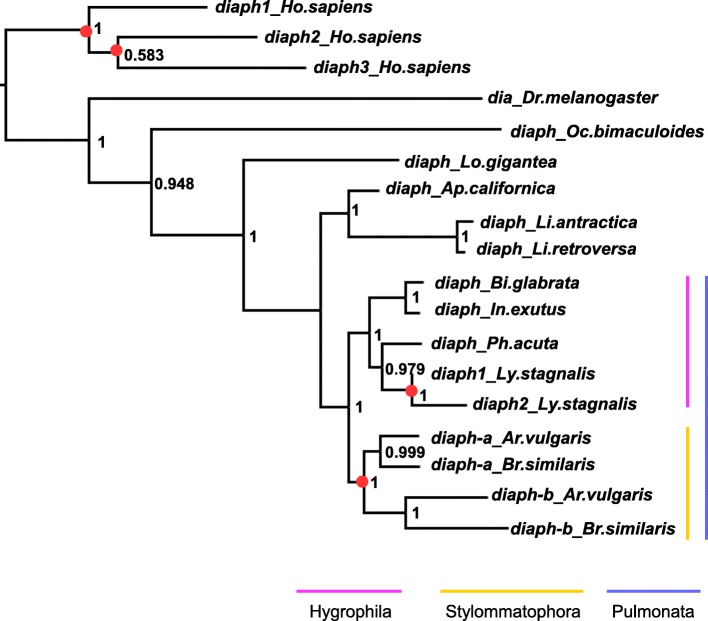
Molecular phylogeny of *diaph* genes. Gene duplication was shown by red circles. Abbreviations are as follows. *Homo sapiens; Ho.sapiens, Drosophila melanogaster; Dr.melanogaster, Octopus bimaculoides; Oc.bimaculoides, Lottia gigantea; Lo.gigantea, Aplysia californica; Ap.californica, Limacina antractica; Li. antractica, Limacina retroversa; Li.retroversa, Biomphalaria glabrata; Bi.glabrata, Indoplanorbis exustus; In.exustus, Physella acuta; Ph. acuta, Lymnaea stagnalis; Ly.stagnalis, Arion vulgaris; Ar.vulgaris, Bradybaena similaris; Br.similaris*

Two *diaph* genes in *Lymnaea* genome suggests that the *B. similaris diaph* gene is also duplicated, with the expression of one being downregulated. Therefore, we carefully examined maternal transcripts of *Bsdiaph* (*diaph*) in a wild strain and found two types of *Bsdiaph* transcript, between which we disntinguich as *Bsdiaph-a* (TRINITY_DN180102_c0_g1) and *Bsdiaph-b* (TRINITY_DN171781_c1_g2)*.* Although their nucleic acid sequences were 71.9% conserved in their protein coding regions, expression of *Bsdiaph-a* didn’t significantly differ between wild and mutant embryos through the EdgeR comparison (*p*-value; 0.47). Molecular phylogenetic analysis indicated independent duplications of *diaph* genes in *Lymnaea* and Stylommatophora (Fig. 2).

**Fig. 3:**
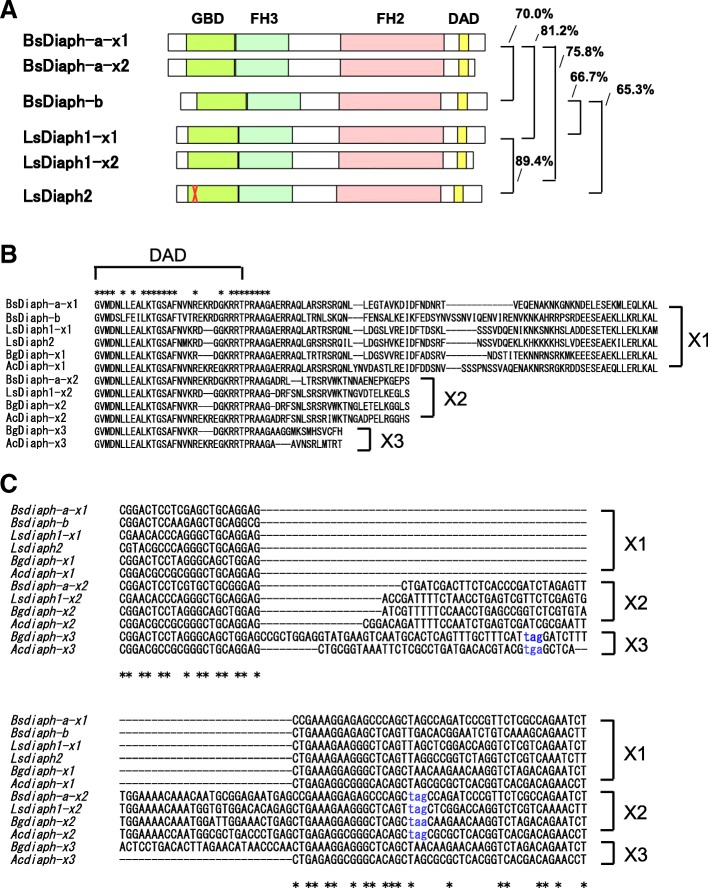
Diaph protein structures. (**a**) Those translated by *Bsdiaph-a* (two splicing variants, × 1 and × 2) and *Bsdiaph-b.* A truncated mutation of LsDiaph-1 is also shown with a red X (from Kuroda et al. 2016). Diaph GBD; Diaphanous GTPase binding domain FH3; Diaphanous FH3 domain FH2; FH2 domain DAD; Diaphanous auto-regulatory domain. (**b**) Comparison of C-terminal amino acid sequences of Diaph between various snail species. (**c**) Alignments of splice variants of *diaph.* Stop codons are shown by blue lower-case letters. Bs, *Bradybaena similaris,* Ls, *Lymnaea stagnalis,* Bg, *Biomphalaria glabrata,* Ac, *Aplysia californica*

Bsdiaph proteins have a Diaphanous GTPase binding domain (GBD), a Formin homology (FH3) domain, an FH2 domain, and a short Diaphanous auto-regulatory domain (DAD) (N- to C-terminal). BsDiaph-a and BsDiaph-b share 70.0% amino acid sequence similarity (Fig. 3a).

In addition, we found splicing variants of *Bsdiaph-a* in the maternal transcriptomes that encode two protein isoforms, Bsdiaph-a-× 1 and Bsdiaph-a-× 2 (Fig. 3a-C). These two isoforms differ in their C-terminal amino acid sequences after the conserved DAD domain, Bsdiaph-a-× 2 being shorter in the C-terminus compared with Bsdiaph-a-× 1 (Fig. 3a). *Bsidiaph-a-× 2* contained additional nucleotide sequences against *Bsidiaph-a-× 1* as in the case of these variants in *B. glabrata* and *A. californica,* and it caused frameshift and an earlier stop codon (Fig. 3c). Other variants which were found in the genome-sequenced gastropods, *B. glabrata* and *A. californica,* e.g. *diaph-× 3,* were not found in our data sets.

### Expression of *Bsdiaph-a* and *Bsdiaph-b*

We identified a gene with two noteworthy features; (a) its maternal transcript level was greatly reduced in the mutant strain compared with the wild strain and (b) *B. similaris* expresses two different types of transcripts, *Bsdiaph-a* and *Bsdiaph-b,* with the expression of the latter likely being down-regulated in the mutant. Therefore, we further examined expression profiles of the two genes by adding data obtained from juveniles and from adult digestive glands in both wild and mutant strains (Additional file 2).

**Fig. 4:**
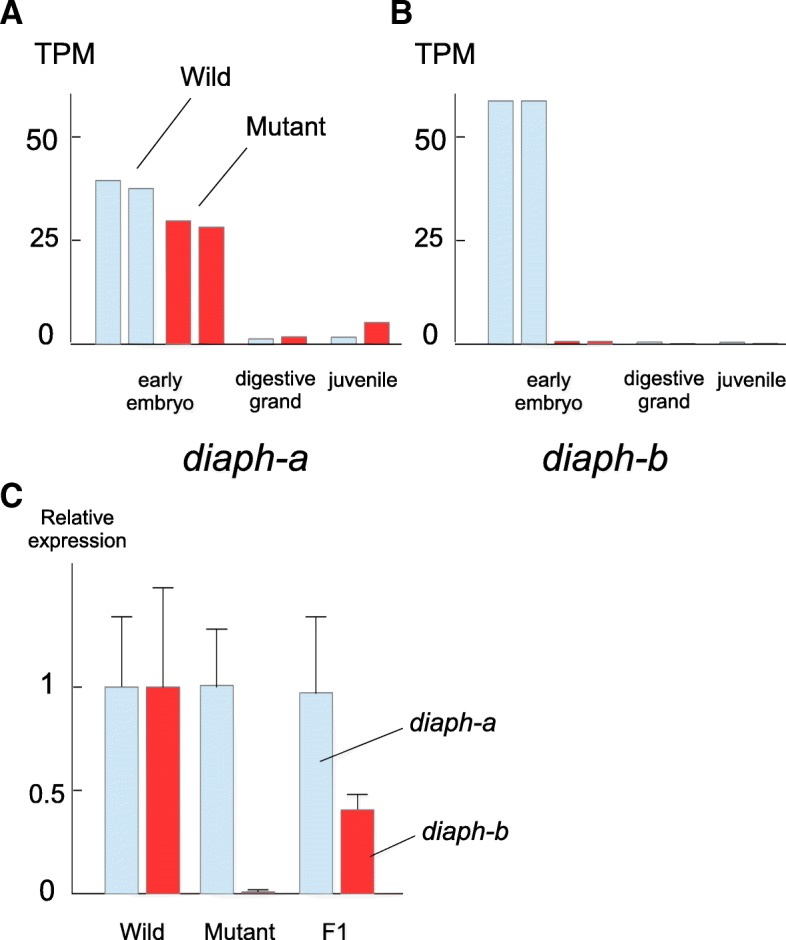
(**a**), (**b**) Comparison of expression levels of *Bsdiaph-a* (left) and *Bsdiaph-b* (right) between wild-type and mutant strains. Levels are shown in early embryos, juveniles, and digestive glands, based on mRNA quantity (TPM, tags per million). Blue bars indicate the TPM score of the wild-type and red bars denote that of the mutant strain. (**c**) Quantification of *Bsdiaph-a* and *Bsdiaph-b* in the early embryos from wild, mutant and F1 strains by qRT-PCR (*n* = 3). Standard error was shown by error bars

The TPM (tags per million) score of *Bsdiaph-a* mRNA was comparable between wild and mutant strains, although slightly lower in the mutant in early-stage embryos (nearly 80% of wild strain) (Fig. 4a). Because *Bsdiaph-a* mRNA is a maternal transcript, its TPM score decreased in juveniles and in adult digestive gland. In contrast, the TPM score of *Bsdiaph-b* differed considerably between wild and mutant strains. The number of *Bsdiaph-b* transcripts in early mutant embryos was less than 1% that of the wild strain (Fig. 4b). The *Bsdiaph-b* transcripts was few in number in juveniles and adult digestive glands of both strains. These results indicate that the expression of *Bsdiaph-b* is maternally suppressed in mutants. On the other hand, maternal expression of *Bsdiaph-a* is normally regulated in both wild and mutant strains. This result was also validated by quantification through qRT-PCR methods. Figure 4c shows expression level of *Bsdiaph-a and Bsdiaph-b* from early embryos of wild, racemic and F1 strains with triplicate biological samples through qRT-PCR. Relative expression level of *Bsdiaph-a* was comparable among three strains as the result of quantification by RNA-Seq data. On hand expression of *Bsdiaph-b* from embryos of the racemic mutant strain was highly reduced and those from F1 strain was lower than those from the wild strain.

### *Bsdiaph-b* transcript is not frame-shifted

In sinistral mutant strain of *Lymnaea stagnalis*, *Lsdiaph2* transcribes mRNA in which a single-base deletion occurs at position 50 of the coding region, which causes a frame-shift to produce truncated diaphanous protein consisting of only the N-terminus (Fig. 3a). To determine whether this is the case in *Bsdiaph-b,* we carefully examined maternal transcripts of *Bsdiaph-b* from mutant embryos, juvenile, and adult digestive gland (Table 1).

**Fig. 5:**
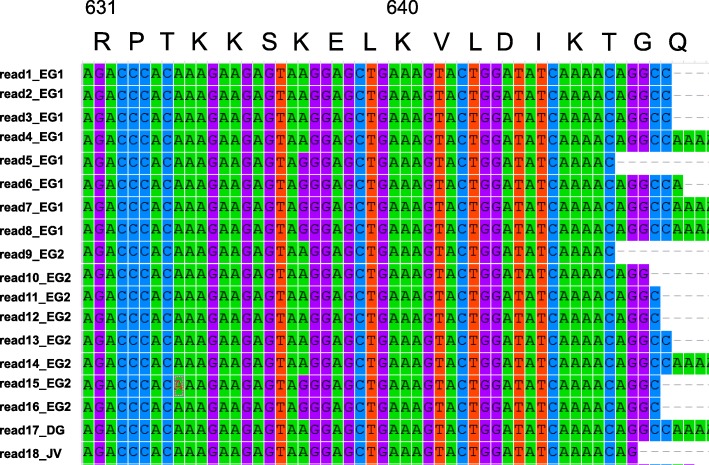
Alignment of nucleotide sequences of *Bsdiaph-b* mRNA obtained by RNA-seq reads from eggs (EG), juveniles (JV) and digestive gland (DG) of the mutant strain. There are no deletions in the sequences

Although the central region (55 nucleotides) of *Bsdiaph-b* transcripts showed a few substitutions of nucleotides (Fig. 5), no clear lesions were identified in BsDiaph-b proteins. We found no non-sense mutations, including frame-shifted transcripts that produce truncated proteins. Instead, at least two alleles were found in the reads from a mutant strain (Fig. 5). These alleles were not from sequencing errors because the reads from four independent samples (early-embryo1, early-embryo2, digestive gland and juvenile) contained two alleles. As the racemic strain of *B. similaris* should be homozygotic for the mutated locus ([24] Utsuno and Asami, 2010), we concluded that *Bsdiaph-b* is not mutated.

## Discussion

The present RNA-seq analysis of differential expression of maternal transcripts in wild (dextral cleavage) and mutant strains (dextral or sinistral cleavage) of *Bradybaena similaris* showed that (1) *Bsdiaph* has two types of transcripts, suggesting the presence of two copies of the gene (*Bsdiaph-a* and *Bsdiaph-b*) in the genome; (2) the amount of maternal mRNA of *Bsdiaph-a* was comparable between the two strains, but that of *Bsdiaph-b* was highly reduced in mutant eggs, suggesting that suppression of *Bsdiaph-b* expression is involved in the mutation; (3) the *Bsdiaph-b* transcript is not frame-shifted in mutant eggs, suggesting that the gene itself is normal. Our finding of no mutation at *Bsdiaph-b*, which is only weakly expressed in the progeny of the racemic mutant, suggests the presence of some regulatory mechanism that is responsible for polarity determination for viable spiral cleavage, other than duplicated *diaph* homologs. The mutated gene may be an upstream trans-regulator or a distantly located cis-element from the locus of *Bsdiaph-b* because of heterogeneity of the sequence of *Bsdiph-b* from mutant snails.

*Lymnaea stagnalis* duplicated *diaph* into tandem-aligned *Lsdiaph1* and *Lsdiaph2.* Diaph protein may have pivotal roles in coordinating functions that depend upon actin filaments ([14] Shibazaki et al., 2004). Davison et al. [16] showed that *Lsdiaph1* and *Lsdiaph2 mRNA* are distributed unevenly into one of the cells in the 2-cell embryo (dextral), while Kuroda et al. [17] found an even distribution*.* This apparent contradiction probably results from the aberrant variability of chiral patterning among mostly non-viable embryos in the *Lymnaea* mutant strain ([12] Utsuno et al. 2011). *Lsdiaph2* mutated to form a functionless protein, so that in the homozygotic *ss* mutant, a reduced amount of Diaph protein is produced. If *diaph* were a single-copy gene with a critical role in early embryonic cleavage, its mutation might disturb the cleavage pattern, resulting in abnormal embryos. Embryonic lethality of mutant animals with a single copy of *diaph* in *Drosophila melanogaster* and *Caenorhabditis elegans*, supports this idea ([32] Castrillon and Wasserman, 1994, [33] Swan et al., 1998). On the other hand, when the gene becomes duplicated and one of the two copies mutates, spiral cleavage may proceed enabling viable embryogenesis. However, only a small portion of siblings survive to hatch as the sinistral in this case of the *Lymnaea* mutant ([24] Utsuno and Asami, 2010).

*Diaph* in *Bradybaena similaris* is also duplicated. Our expression analysis indicated reduced expression of *Bsdiaph-b* and normal expression of *Bsdiaph-a* in early embryos of the mutant strain. This transcriptional phenotype is similar to the *Lymnaea* mutant*.* Although the differential expression of *Bsdiaph-a* and *Bsdiaph-b* implies their diversification, products from *Bsdiaph-a* could compensate for loss of crucial functions of Diaph protein in early embryogenesis when *Bsdiaph-b* expression is reduced*.*

Despite their similarity in *diaph* homologs’ expression patterns, the mutant phenotypes differ between *L. stagnalis* and *B. similaris*. The racemic mutant in *B. similaris* generates both dextral and sinistral progeny, while offspring of the *Lymnaea* mutant develop into the sinistral only, although the majority of siblings fail in early embryogenesis in both cases. One possible explanation for these phenotypes is gleaned from the difference between null and leaky mutants. *Lsdiaph2* of the *Lymnaea* mutant strain is frame-shifted and is considered a null mutation, while no clear lesions were found in the sequence of *Bsdiaph-b*. A slight amount of *Bsdiaph-b* was detected in early embryos from the racemic mutant of *B. similaris,* even though expression was highly reduced (Fig. 4). Although our RNA-seq data doesn’t establish absence or presence of proteins, it is possible that the reduced amount of the gene product from *Bsdiaph-b* is produced in racemic mutant embryos, and it partly compensates for their phenotypes. If the gene product of *Bsdiaph-b* is crucial for the change of cleavage direction from sinistral to dextral, and the racemic mutant of *B. similaris* is a leaky mutant, their siblings become mixture of both dextral and sinistral individuals.

Another hypothesis is that the default chiral directionality without functional protein products of duplicated *diaph* differs between the dextral Lymnaeidae clade that relatively recently evolved by reversal from the sinistral ancestor in the Hygrophila ([16] Davison et al. 2016) and the other *Baradybaena* clade of the dextral family Camaenidae that has long retained dextrality in the Stylommatophora ([34] Köhler and Criscione, 2015). In *L. stagnalis*, spiral cleavage becomes somewhat reversed, and a fraction of sinistrally developing siblings survive to hatching. In *B. similaris,* on the other hand, no clear direction could be determined for spiral cleavage. Early embryonic cells could perform sinistral cleavage by lacking a determinant to be dextral in *Lymnaea* mutant, while the defect of polarity regulation in embryonic cells results in directionally randomized cleavage in the *Bradybaena* mutant (Fig. 1b).

Recent reports have highlighted the importance of cellular chirality for determination of left-right handedness of several organs during embryogenesis ([35] Taniguchi et al., 2011, [36] Inaki et al., 2016). In *Drosophila melanogaster*, embryonic gut, male genitalia and adult hind gut are directionally rotated organs, and their proper morphogenesis to right or left is controlled by the chirality of each cell. One of the functions of formin proteins is control of cellular chirality through construction of radial fibers of actin filaments ([37] Tee et al., 2016). Actin filaments are important for spiral cleavage ([13] Shibazaki et al., 2004). Between dextral *Lymnaea* and sinistral *Physa*, early blastomeres are reversed in cellular chirality. The cortical layer of blastomere even at the first cell division moves clockwise in the dextral group but counterclockwise in the sinistral group ([38] Meshcheryakov and Beloussov, 1974). Brun-Usan et al. [39] pointed out the importance of directed cellular chirality for proper spiral cleavage by computational simulation. The spiral cleavage of progeny from the racemic mutant of *B. similaris* resembles their direction-randomized spiral cleavage with no directed cortical rotation.

In gastropods that copulate for reproduction, left-right reversal results in genital mismatch with the wild type because the genital orifix is located in the body side instead of the midline. Thus, population fixation for reversal contributes to reproductive isolation, especially in stylommatophoran pulmonates that copulate simultaneously reciprocally ([40] Gittenberger, 1988; [23] Asami et al., 1998; [20] Ueshima and Asami, 2003).

Left-right reversal also functions for surviving or avoiding predation in some environments ([41] Hoso et al., 2007, [21] Hoso et al., 2010, [42] Danaisawadi et al., 2016). Southeast Asian snail-eating snakes specialize in predation on the dextral majority in snails and avoid or fail to capture sinistrals. The advantage of sinistrality for avoidance of predation accelerated speciation through left-right reversal from dextral ancestors in the range of these snakes. In such cases, left-right reversal by a single gene may have played a critical role for speciation. However, molecular genetic mechanisms for their developmental reversal have not been identified. Left-right reversal of spiral cleavage via mutation or transcriptional reduction of one *diaph* might have been involved for the evolution of reversed populations/species. If the presence of two *diaph* genes by duplication might have been advantageous for evolution of the reversal in pulmonate snails, this should be considered an exaptation.

The evolution of left-right reversal requires population fixation for reversal that permits equivalent or superior Darwinian fitness relative to the wild type ([18] Okumura et al., 2008). However, in either cases of the mutants of *L. stagnalis* ([11] Asami et al. 2008, [12] Utsuno et al. 2011) or *B. similaris* ([24] Utsuno and Asami, 2010), most of sibling embryos exhibit aberrant reversals and fail to hatch. Thus, these mutations are to be eliminated by internal selection and cannot give rise to sinistral populations or species. In contrast, for example, the wild-type sinistral embryos of the hygrophilan pond snails *Physa* and *Biompharalia* typically perform reversed mirror-image cleavage compared with the wild-type dextral cleavage of *Lymnaea* ([43] Crampton, 1894, [44] Camey and Verdonk, 1970) unlike the “sinistral” mutant of *L. stagnalis*. This means that we do not yet know about mutations and genetic mechanisms that could have generated reversed snail species from the dextral or sinistral ancestral populations. Our study accordingly has important implications to explore how reversed populations/species have recurrently evolved if mutation for the *diaph* function has been involved.

## Conclusions

Maternal expression of one of the *diaph* homolog (*Bsdiaph-b*) is most reduced in progeny of the racemic mutant of the stylommatophoran pulmonate *Bradybaena similaris*, which generates both dextral and sinistral progeny. The other *diaph* homolog (*Bsdiaph-a*) is equivalently expressed between the wild-type dextral and mutant. These homologs originated by duplication in the stylommatophoran ancestor. However, no clear lesion is present in the *diaph* gene itself, unlike the case of frame-shift mutation which results in incomplete sinistral development in the hygrophilan pulmonate *Lymnaea stagnalis*. Our results suggest that a regulatory mechanism controls the left-right polarity of spiral cleavage. Our study provides a new molecular basis that promotes further studies for understanding of the evolution of spiral cleavage in gastropods which frequently evolved left-right reversal.

## Additional files

Additional file 1: **Table S2.** IDs of genes for the molecular phylogenetic analysis. (DOC 20 kb)

Additional file 2: **Table S1.** RNA-seq reads by Hiseq4000 of *Bradybaena similaris.* (XLS 9 kb)
