# Supplementary material for: Heterochirality results from reduction of maternal diaph expression in a terrestrial pulmonate snail
Source: Zoological Lett. 2019 Jan 10;5:2. doi: 10.1186/s40851-018-0120-0 (PMC6329061; doi:10.1186/s40851-018-0120-0)
Supplement: Supplementary file 1 — Table S2. IDs of genes for the molecular phylogenetic analysis. (DOC 20 kb) [file 40851_2018_120_MOESM1_ESM.doc]

| Species | Gene name | ID |
| --- | --- | --- |
| *Homo sapiens* | *diaph-1* | NM_005219.4 |
| *Homo sapiens* | *diaph-2* | NM_006729.4 |
| *Homo sapiens* | *diaph-3* | NM_001042517.1 |
| *Drosophila melanogaster* | *dia* | NM_057633.5 |
| *Octopus bimaculoides* | *diaph* | XM_014934596.1 |
| *Lottia gigantea* | *diaph* | XM_009051963.1 |
| *Aplysia californica* | *diaph* | XM_013088861.1 |
| *Limacina retroversa* | *diaph* | GBXC01026131.1 |
| *Limacina antarctica* | *diaph* | GDRM01025693.1 |
| *Biomphalaria glabrata* | *diaph* | XM_013217703.1, XM_013222366.1 |
| *Indoplanorbis exustus* | *diaph* | KX387876.1 |
| *Physella acuta* | *diaph* | KX387875.1 |
| *Lymnaea stagnalis* | *diaph1* | AOV18873.1 |
| *Lymnaea stagnalis* | *diaph2* | AOV18871.1 |
| *Arion vulgaris* | *diaph-a* | HACG01040862.1 |
| *Arion vulgaris* | *diaph-b* | HACG01040866.1 |
| *Bradybaena similaris* | *diaph-a* | MK061308 |
| *Bradybaena similaris* | *diaph-b* | MK061307 |
